# Magnetic ordering and dynamics in monolayers and bilayers of chromium trihalides: atomistic simulations approach

**DOI:** 10.1038/s41598-024-75501-2

**Published:** 2024-10-26

**Authors:** S. Stagraczyński, P. Baláž, M. Jafari, J. Barnaś, A. Dyrdał

**Affiliations:** 1 Faculty of Physics and Astronomy, Adam Mickiewicz University in Poznań, ul, Uniwersytetu Poznańskiego 2, 61-614 Poznań, Poland; 2 FZU – Institute of Physics of the Czech Academy of Sciences, Na Slovance 1999/2, 182 21 Prague 8, Czech Republic

**Keywords:** Spintronics, Magnetic properties and materials

## Abstract

We analyze magnetic properties of monolayers and bilayers of chromium iodide, $\hbox {CrI}_3$, in two different stacking configurations: AA and rhombohedral ones. Our main focus is on the corresponding Curie temperatures, hysteresis curves, equilibrium spin structures, and spin wave excitations. To obtain all these magnetic characteristic, we employ the atomistic spin dynamics and Monte Carlo simulation techniques. The model Hamiltonian includes isotropic exchange coupling, magnetic anisotropy, and Dzyaloshinskii-Moriya interaction. As the latter interaction is relatively weak in pristine $\hbox {CrI}_3$, we consider a more general case, when the Dzyaloshinskii-Moriya interaction is enhanced externally (e.g. due to gate voltage, mechanical strain, or proximity effects). An important issue of the analysis is the correlation between hysteresis curves and spin configurations in the system, as well as formation of the skyrmion textures.

## Introduction

Van der Waals (vdW) magnetic materials are currently of great interest due to expected applications in two-dimensional (2D) nanoelectronics and spintronics. These materials are built from weakly coupled 2D monolayers, and therefore they can be relatively easily obtained in the multilayered form^1^, down to a single monolayer. The discovery of ferromagnetism in monolayers of $$\hbox {CrI}_3$$^2^ and $$\hbox {Cr}_2$$$$\hbox {Ge}_2$$$$\hbox {Te}_6$$^3^ initiated an enormous interest in magnetic 2D vdW crystals^4–6^. There are several groups of well-known magnetic vdW materials, including chromium trihalides $$\hbox {CrX}_3$$ (X = I, Cl, Br)^2,7^, metal tribromides $$\hbox {MBr}_3$$ (M = Mn, Cu, Fe, V), chromium-based ternary tellurides $$\hbox {Cr}_2$$$$\hbox {XTe}_6$$ (X = Ge, P)^3^, transition metal dichalcogenides $$\hbox {MnX}_2$$ and $$\hbox {VX}_2$$ (X = S, Se, Te) in two different phases denoted as 2H and 1T,^8–10^, and also others. The number of known 2D magnetic van der Waals materials is growing rapidly, and they have various structural, electronic, magnetic, and transport properties. There is currently extensive search for new materials, with new properties, and new perspectives for applications, as these materials seem to be ideal for building ultra-thin (of atomic scale) nanoelectronic devices (like spin valves or nonvolatile memory elements) for, e.g., quantum computing, spintronics, magnonics, opto-electronics, and others^4,11^. However, magnetic properties of most of the known 2D van der Waals systems still require further investigations. The magnetic ground state of 2D vdW crystals depends, among others, on their crystallographic phase, stacking geometry, substrate, and on possible twisting of adjacent monolayers. In many cases, the magnetic ground state survives only at low temperatures, while at higher temperatures the systems become paramagnetic. Therefore, much efforts are focused on searching for materials with critical temperatures above the room temperature.

Another group of 2D materials are layered molecular magnets. Indeed, a number of molecular magnets have been synthesized in the monolayer and bilayer forms^12–14^. Both 2D vdW materials and 2D molecular magnets offer a unique opportunity to test various theoretical models of magnetic interactions and magnetic physical phenomena in two dimensions. As for the interactions, this includes exchange symmetric coupling, Dzyaloshinskii–Moriya Interaction (DMI), magnetic anisotropy, higher-order interactions, like two-ion magnetic anisotropy (or Kitaev term), and also others^7,15^. Many of these materials have the ground state spin orientation perpendicular to the monolayers or bilayers. In some cases, the magnetic anisotropy enforces in-plane spin orientation. Moreover, these materials also allow to observe various topological phases and phase transitions, like strictly 2D skyrmions or topological Berezinskii-Kosterlitz-Thouless phase transitions^12,16^.

Among various groups of vdW materials, there are materials which are promising for applications, like for instance transition metal dichalcogenides, especially Vanadium dichalcogenides $\hbox {VX}_2$ (X = S, Se, Te)^8–10^, and chromium trihalides, e.g., chromium iodide and chromium chloride, $\hbox {CrI}_3$ and $\hbox {CrCl}_3$^2,7,15^. In this paper we will focus on the latter group, specifically on $\hbox {CrI}_3$. Monolayers of this material are ferromagnetic, with the magnetic moments oriented perpendicularly to the layer plane^2,17^. This material is very interesting for fundamental research^18,19^. However, the corresponding Curie temperature is around 40 K^2^, which is too low for most of practical applications. In the bilayer form, the two monolayers are coupled antiferromagnetically. However magnetic properties can be externally tuned by electric field^20,21^ or mechanical strains^22–28^, and interlayer coupling can be even changed from antiferromagnetic to ferromagnetic one^29^. Moreover, magnetic properties of $\hbox {CrI}_3$ in a multilayer form depend on the stacking geometry^30–32^. This material seems to be ideal for testing various theoretical models of spin configuration, including topological skyrmion textures^33–36^, as well as excited states (spin waves) which display certain topological features^7,37–41^. It was pointed out^36^, that DMI in $\hbox {CrI}_3$ is too small to generate skyrmion textures. However, it was also predicted that DMI in Janus monolayers of chromium trihalides Cr(I,X)$_3$ can be significantly larger than that in $\hbox {CrI}_3$, and can generate magnetic skyrmion textures^34^. Therefore, we assume a more general case, which admits larger values of DMI (e.g. in Janus structures).

**Figure 1:**
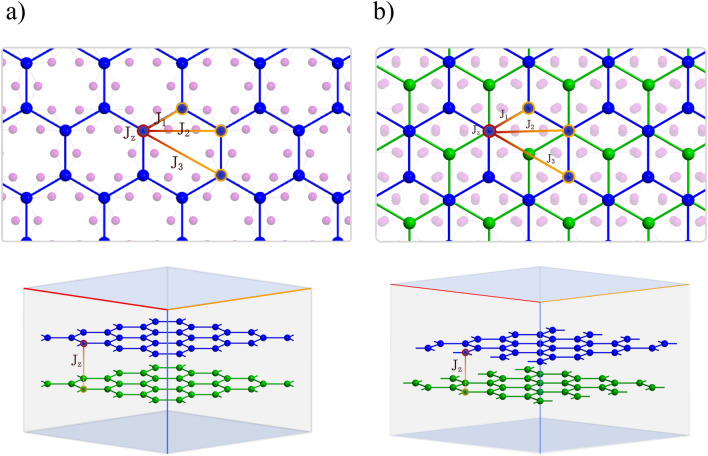
Atomic structure of the bilayers of $\hbox {CrI}_3$ in the AA stacking of layers without offset (**a**), and in the rhombohedral stacking, where the offset is half of the unit cell vectors $\textbf{a} / 2$, $\textbf{b} / 2$ (**b**). The Cr atoms of the top (bottom) monolayer are indicated in blue (green). The top panels present the top views, while the bottom panels show the side views. Note, the top part of (**a**) shows effectively atomic structure of a monolayer. The I atoms are indicated in the top parts in pink, while for clarity reasons they are not indicated in the bottom parts. The intralayer and interlayer exchange couplings are also marked in the top and bottom parts, respectively.

In this paper we analyze magnetic properties of $\hbox {CrI}_3$, and to do this we employ simulation methods like atomistic spin dynamic (ASD) technique and Monte Carlo simulations^42–44^. From this we determine such magnetic properties like Curie temperature, hysteresis curves, (quasi)equilibrium spin configurations, or excited states (spin waves). We limit considerations to monolayers and bilayers of $\hbox {CrI}_3$. As some results on the monolayers are already available in the literature, much less work has been done on the bilayer structures of $\hbox {CrI}_3$. Atomic structure of the bilayers of $\hbox {CrI}_3$ is shown in Fig. 1 for two different stacking of the monolayers. This figure shows top and side views, and atomic structure of a single monolayer is effectively shown by the top part of Fig. 1a.

## Model and methods

### Structure and spin Hamiltonian

Magnetic properties of chromium trihalides remarkably depend on the number of monolayers in the sample, and also on the geometry and offset of their stacking. This difference is especially significant for small numbers of layers in the system. The most stable stacking geometries are the AA and Rhombohedral ones, see Fig. 1. The following analysis is limited to both monolayers and bilayers of $\hbox {CrI}_3$. Magnetic moments in $\hbox {CrI}_3$ are predominantly localized on the chromium atoms, while contributions from iodine atoms are small and may be omitted. However, the iodine atoms contribute to exchange interactions between the magnetic Cr atoms (e.g. via superexchange), and thus have a significant impact on magnetic properties of $\hbox {CrI}_3$. The Cr atoms in an individual monolayer form a hexagonal (honeycomb) lattice, and positions of these atoms have been taken from the unit cell of bulk $\hbox {CrI}_3$.

Accordingly, magnetic properties of $\hbox {CrI}_3$ will be described by an effective spin Hamiltonian attributed to the corresponding hexagonal lattice of Cr atoms, which we split into two terms,

1 $$\begin{aligned} {\mathcal {H}}= {\mathcal {H}}_0 + {\mathcal {H}}_\textrm{DM}, \end{aligned}$$

where ${\mathcal {H}}_0$ includes the exchange interactions, magnetic anisotropies, and the Zeeman energy in an external magnetic field ${\textbf{B}}$. The second term, ${\mathcal {H}}_\textrm{DM}$, takes into account antisymmetric exchange, known also as Dzyaloshinskii-Moriya Interaction (DMI).

Assuming isotropic exchange interactions, we write the first term in the form

2 $$\begin{aligned} {\mathcal {H}}_0 = -\sum \limits _{ij} J_{ij} {{\textbf{S}}}_i \cdot {{\textbf{S}}}_j - k\sum \limits _i \left( S^z_i\right) ^2 - \mu _S \sum \limits _{i} {{\textbf{B}}}\cdot {{\textbf{S}}}_i, \end{aligned}$$

where the sum over *i* and *j* means the summation over lattice sites. Here, the positive exchange parameters $J_{ij}$ correspond to ferromagnetic (FM) coupling, whereas negative ones correspond to antiferromagnetic (AFM) coupling. In the following we take nonzero exchange parameters between nearest-neighbours, second-nearest-neighbours and third-nearest-neighbors, denoted respectively as $J_1$, $J_2$, and $J_3$. In a more general case the exchange parameters can be anisotropic. The second term describes the magnetic anisotropy. Here, positive constant *k* corresponds to the easy-axis anisotropy along the *z*-direction (normal to the layer), while negative *k* corresponds to the easy-(*xy*)plane anisotropy. Furthermore, $\mu _s$ is the atomic magnetic moment of Cr atoms. Here, and in the following, if not otherwise stated, the vector ${\textbf{S}}$ is a unit vector along the orientation of a local spin moment^42^. Accordingly, one needs to adjust the exchange parameters, which usually are related to spin Hamiltonian, where ${\textbf{S}}$ is the spin operator.

The second term in Eq. (1) includes the Dzyaloshinskii-Moriya interactions (or alternatively antisymmetric exchange). Existence of nonzero components of this interaction depends on symmetry of the system. In van der Waals materials one can easily tune externally the symmetry and thus also the components of DMI, e.g., by an external mechanical strain or by electric field normal to the layers. In the case under consideration, the DMI Hamiltonian can be written in the form^15^

3 $$\begin{aligned} {\mathcal {H}}_\textrm{DM} = -\sum \limits _{\left\langle i, j \right\rangle } {{\textbf{d}}}^{NN}_{ij} \cdot \left( {\textbf{S}}_i \times {\textbf{S}}_j \right) -\sum \limits _{\left\langle \left\langle i, j \right\rangle \right\rangle } {\textbf{d}}^{NNN}_{ij} \cdot \left( {\textbf{S}}_i \times {\textbf{S}}_j \right) , \end{aligned}$$

where the first term includes interaction between intralayer nearest-neighbours (NN), while the second term between next nearest-neighbours (NNN). Here, ${{\textbf{d}}}^{NN}_{ij}$ and ${{\textbf{d}}}^{NNN}_{ij}$ are the corresponding Dzyaloshinskii-Moriya vectors^15^. This vector for NN has the following form: ${\textbf{d}}^{NN}_{ij} = d^{NN}_{xy} \hat{\textrm{z}} \times \hat{\textrm{l}}_{ij} + \tau _{ij}^{NN} d^{NN}_z \hat{\textrm{z}}$, where $\hat{\textrm{z}}$ is a unit vector along the axis *z* (normal to the layer), $\hat{\textrm{l}}_{ij}$ is the unit vector oriented from the site *i* to site *j*, while $d^{NN}_{xy}$ and $d^{NN}_z$ are parameters and $\tau _{ij}^{NN} =1 (-1)$ for the AB (BA) link. In turn, in the second term ${\textbf{d}}^{NNN}_{ij} = \tau ^{NNN}_{ij} \left( d_{xy}^{NNN} \hat{\textrm{l}}_{ij} + \nu _{ij} d_z^{NNN} \hat{\textrm{z}} \right)$, with $\tau ^{NNN}_{ij} = +1$ for AA-link and $-1$ for BB-link, while $\nu ^{NNN}_{ij} = \pm 1$ and alternates its sign on the consecutive NNN links of a chosen site^15^. Finally, we note, that such terms like anisotropic exchange, biquadratic exchange, and higher-order anisotropy terms (e.g., two-ion anisotropy or Kitaev term) have been omitted in the Hamiltonian assumed here.

An important issue in the following numerical calculations is the appropriate choice of the parameters in the above assumed Hamiltonian. In the relevant literature, these parameters have been calculated by several methods, and the most reliable ones are those based on Hubbard-like microscopic models and DFT calculations. For the intrinsic (in pristine systems) exchange and anisotropy parameters we use those determined in Ref. ^7^. Accordingly, for the intralayer exchange constants, adapted to the definitions used in the relevant numerical code (here the Vampire code), we assume in the following: $J_1 = 4.020$meV, $J_2 = 0.643$meV, and $J_3 = 0.016$meV. In turn, for the interlayer exchange in bilayers we assume $J_z = J_2$ for ferromagnetic and $J_z = -J_2$ for the antiferromagnetic interlayer coupling. Additional parameters are the magnetic anisotropy constant $k = 0.109$ meV, and the chromium magnetic moment $\mu _{Cr} = 3.36 \mu _B$. As concerns the interlayer exchange, this parameter is usually very small in vdW materials, and we assumed it as an approximated value obtained from DFT calculations and adapted to the definition used in the Vampire code. All the above parameters are treated as constant in this paper. In turn, for the intrinsic DMI parameters we use their values obtained in Ref. ^15^ from DFT calculations. Accordingly, the nearest-neigbours DMI parameters, $d^{NN}_{xy}$ and $d^{NN}_z$ vanish in pristine systems. However, the DMI parameters between the next-nearest-neighbours are finite, though rather small: $d_z^{NNN}=-8.8\mu \textrm{eV}$ and $d_{xy}^{NNN}=73.0\mu \textrm{eV}$^15^. All the DMI parameters can be relatively easily tuned in experiments (or even generated in case they are zero in a pristine system) by several methods. For instance, they can be tuned by external electric field (due to gate voltage), or by external mechanical strain. Another method used to enhance the DMI interaction in monolayers or bilayers relies on the proximity effects in heterostructures, where a monolayer (bilayer) is deposited (or covered) on another van der Waals material. This method is especially efficient when the cover layer (substrate) is ferroelectric^45^. Moreover, a relatively strong DMI occurs in the so-called Janus chromium trihalides Cr(I,X)$_3$ (where X stands for atoms different from I atoms)^34^. Therefore, bearing in mind that the DMI parameters can be relatively easily tuned in experiments, in the following we will do calculations for various values of the DMI parameters, which will be indicated in each figure. The other parameters, especially the exchange constants and magnetic anisotropy, will be fixed for all calculations, unless otherwise stated.

### Numerical procedures

Our numerical calculations are based on the atomistic spin dynamics (ASD) method implemented into the Vampire code^42^. This method allows for taking into account all details of the system geometry, and also for keeping insight into behaviour of the spin moments during the simulation process. The ASD simulations are carried out by solving a coupled system of atomistic stochastic (at finite temperature) Landau-Lifshitz-Gilbert (LLG) equations,

4 $$\begin{aligned} \frac{\partial {\textbf{S}}_i}{\partial t} = -\frac{\gamma }{1 + \alpha ^2} \left[ {\textbf{S}}_i \times {\textbf{B}}^\textrm{eff}_i + \alpha {\textbf{S}}_i\times ({\textbf{S}}_i \times {\textbf{B}}^\textrm{eff}_i )\right] , \end{aligned}$$

where $\gamma$ is the gyromagnetic ratio, $\alpha$ stands for the Gilbert damping parameter, and ${\textbf{B}}^\textrm{eff}_i$ is the effective magnetic field acting on the spin at site *i*. This effective field ${\textbf{B}}^\textrm{eff}_i$ consists of two terms: one is deterministic and is related to the spin Hamiltonian, while the second one is a stochastic term, that is determined by thermal fluctuations,

5 $$\begin{aligned} {\textbf{B}}^\textrm{eff}_i = \frac{-1}{M_s} \frac{\partial {\mathscr {H}}}{\partial {\textbf{S}}_i} + \mathbf {\xi }^\mathrm{(th)}_i. \end{aligned}$$

In the low energy regime, the stochastic term, $\mathbf {\xi }^\mathrm{(th)}_i$ is usually modelled by the Gaussian thermal noise.

To verify the results on spin dynamics obtained by the atomistic LLG equations, we also used other methods, like the method based on the spin-spin correlation function and the Monte Carlo simulation technique. We find a very good agreement between the results obtained by the atomistic LLG equations and those obtained by the Monte Carlo simulations. As the former technique is more appropriate for dynamical properties, the latter one is more convenient for static properties. However, the static properties are also accessible from spin dynamics, but require much higher computational efforts. In addition, to study dynamical properties we also use the atomistic spin dynamics approach implemented into the Uppsala ASD code^46^.

An important step in the simulation process, especially when simulating hysteresis loops, critical temperatures, or magnon dispersion relations, is the preparation of relevant initial spin configuration. To do this we use the field-cooling procedure. We start this procedure always with the random spin state at a high temperature (above the Curie temperature). After performing one million of Monte Carlo equilibration steps (or time steps for LLG equation), the temperature becomes decreased nearly adiabatically to the minimum temperature, i.e. to the temperature, at which the required simulations are to be performed, e.g. 15 K in most of our simulations of the hysteresis loops.

More specifically, in the simulation of the hysteresis curves, the initial temperature of the system was 80 K. Then 1,000,000 equilibration steps were performed to reach 15K. Accordingly, $dT = 65K/10^6$ per MC sweep. Additional 20,000 MC equilibration steps were performed at the temperature of 15 K. When cooling the system from 15 K to 0 K (e.g., to observe skyrmions), then additional 1000,000 MC steps were performed with $dT = 15K/10^6$. This procedure was performed for each magnetic field assumed when sweeping the whole hysteresis loop.

Stability of the final simulation processes was verified by calculating the corresponding mean torque components. Practically, we performed additional 10,000 LLG or MC steps. At the beginning (well below 5000 steps), there were very weak and decaying oscillations of the torque components, while for a larger number of simulation steps, there was no signature of the oscillations, so we could safely assume that the equilibrium (or quasi-equilibrium) state was reached.

In the following we will apply the above mentioned methods to determine the Curie (Néel) temperature, as well as hysteresis loops at a finite (nonzero) temperature. The simulations will be performed for monolayers, as well as for bilayers in two different stacking geometries, i.e., in the AA and rhombohedral configurations. In addition we will also consider dynamical properties of the systems under consideration, especially spin wave excitations (magnons). In all the numerical simulations we used the periodic boundary conditions, and the sample size was 20 nm$\times$20 nm when simulating the hysteresis loops and critical temperatures, while it was 120 nm$\times$120 nm for the simulations of magnon dispersion curves.

## Results

### Critical temperatures

The reported experimental data indicate on a ferromagnetic (FM) ground state in $\hbox {CrI}_3$ monolayer^2^. However, the bilayer of $\hbox {CrI}_3$ was reported to display antiferromagnetic (AFM) interlayer exchange coupling^2^, so such bilayers should reveal not only features typical of ferromagnets, but also some properties typical of antiferromagnets. In this context it is worth to note that the interlayer coupling can be tuned experimentally (including also sign reversal), e.g., by an external gate voltage^20,21^ or strain^23,24,26–29^. Therefore, one of our main objectives in the case of bilayers is to investigate the impact of the layer stacking (visualized in Fig. 1) and character of interlayer exchange coupling (FM or AFM) on the critical temperature. Moreover, in antiferromagnetically coupled bilayers one may expect certain features related to the breakdown of ferromagnetic order within the monolayers and of antiferromagnetic order related to the interlayer coupling (Curie and Néel temperatures).

**Figure 2:**
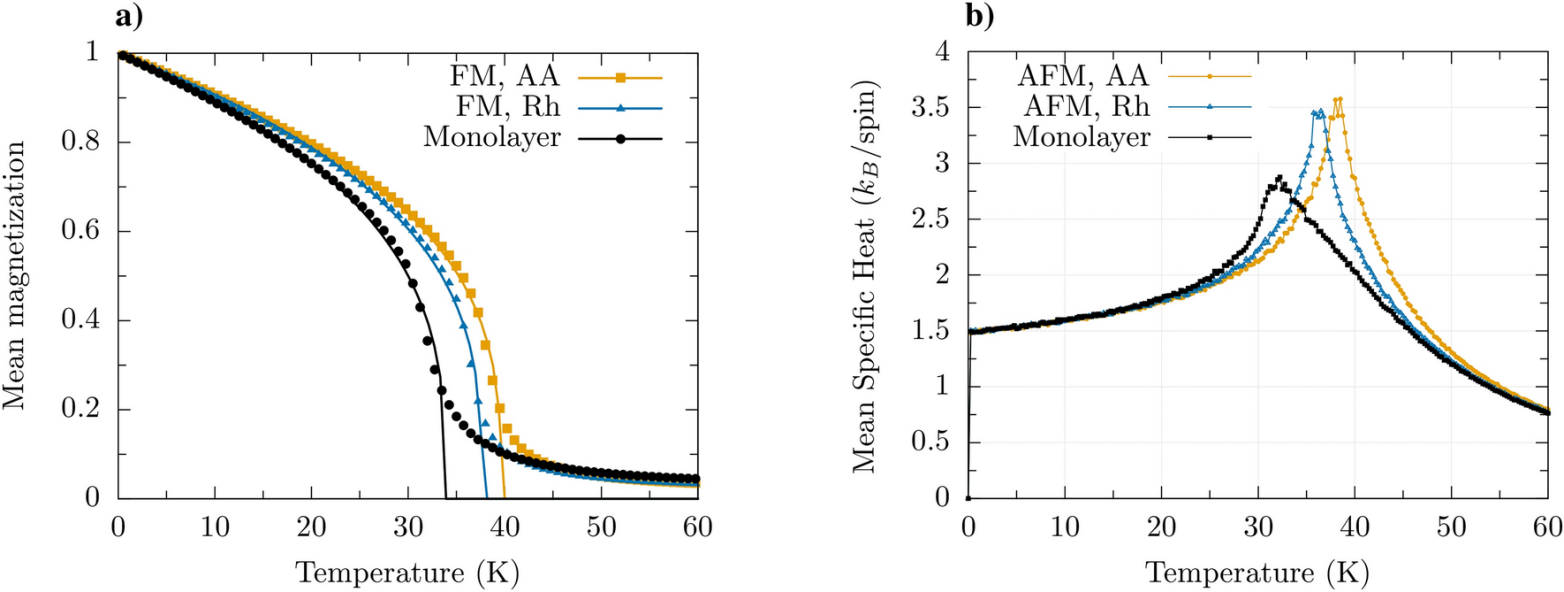
(**a**) Simulated (dots) magnetization vs. temperature for the monolayer and ferromagnetically coupled bilayers in the AA and Rh-stacking. The critical temperatures have been determined from fitting to the formula $(1-T/T_C)^\beta$ (solid lines). From this fitting we determined the Curie temperatures and the exponents $\beta$. For monolayer: $T_C = 33.93$K, $\beta =0.32$; for ferromagnetically coupled bilayer in the Rh stacking: $T_C = 37.64$K, $\beta =0.31$; for ferromagnetically coupled bilayer in the AA stacking: $T_C = 39.64$K, $\beta =0.32$. (**b**) Simulated mean specific heat vs. temperature for the antiferromagnetically coupled bilayers in the AA and Rh-stacking. We have added here the data for the ferromagnetic monolayer, to show that the Curie temperature determined from simulations of the specific heat is equal to that determined from simulations of the magnetization. The critical temperatures are determined by positions of the cusps. The critical temperatures of the bilayers are practically the same as the critical temperatures of the corresponding bilayers with ferromagnetic interlayer coupling. The parameters assumed in simulations: intralayer exchange parameters: $J_1 = 4.020$meV, $J_2 = 0.643$meV, $J_3 = 0.016$meV); interlayer exchange in bilayers: $J_z = J_2$ (**a**) and $J_z = -J_2$ (**b**); magnetic anisotropy $k = 0.109$ meV, and the chromium magnetic moment $\mu _{Cr} = 3.36 \mu _B$. In turn, the DMI parameters: $d^{NN}_{xy} = 0.201$meV and $d^{NN}_z=0$, $d_z^{NNN}=-8.8\mu \textrm{eV}$ and $d_{xy}^{NNN}=73.0\mu \textrm{eV}$.

The simulated data (points) for the monolayer and ferromagnetically coupled bilayers in the AA and Rh stacking geometries are shown in Fig. 2a. To determine the Curie temperatures, the corresponding data have been fitted to the formula $(1-T/T_C)^\beta$, and from this fitting we found $T_C \approx 33.93$ K for the monolayer, $T_C \approx 37.64$ K for the Rh-stacked bilayer, and $T_C \approx 39.64$ K for the AA-stacked bilayer. The Curie temperature for the monolayer is lower than that for the bilayers, and the bilayer in the AA-stacking has Curie temperature higher than that for the Rh-stacking. For the assumed parameters, the DMI has no significant impact on the magnetization vs. temperature profile and on the Curie temperature as follows from comparison of the results with those in the absence of DMI (not shown).

The critical temperatures (both Curie and Néel) also can be determined from magnetic susceptibility or specific heat. In the case of ferromagnetic monolayer and ferromagnetically coupled bilayers, both these methods give results which are consistent with those obtained from the above magnetization analysis. Therefore, to determine the critical temperature in bilayers with antiferromagnetic interlayer coupling, we used the Vampire package^43,44^ to simulate the specific heat as a function of temperature, $C_v = [\left\langle U^2 \right\rangle - \left\langle U \right\rangle ^2]/k_B T^2$, where *U* is the internal energy. The specific heat has a cusp at the critical temperature, and this property is used to determine the Curie temperature, $T_c$, in ferromagnetic systems, and the Néel temperature in antiferromagnets.

The numerical results on the specific heat for bilayers with antiferromagnetic interlayer coupling in the AA and Rh stackings and also for the monolayer are shown in Fig. 2b. The critical temperatures for the bilayers with antiferromagnetic interlayer coupling are clearly seen and are roughly the same as the Curie temperatures in the corresponding bilayers with ferromagnetic interlayer coupling. This is because absolute value of interlayer coupling is remarkably smaller than the intralayer exchange coupling constant $J_1$, and absolute magnitude of the interlayer coupling is assumed the same in bilayers with ferro- and antiferromagnetic interlayer coupling. The curve for ferromagnetic monolayer is presented in Fig. 2b just to show that the methods used in simulations of the results in Fig. 2a,b give the same critical temperatures.

When the interlayer exchange coupling is antiferromagnetic, the total magnetic moment of the bilayer vanishes in the ground state. As already mentioned above, the absolute magnitude of the interlayer coupling is remarkably smaller than the intralayer exchange coupling $J_1$. Thus, with increasing temperature, the antiferromagnetic moment (Néel vector) vanishes first and we have a system of two ferromagnetic layers (with average moments either aligned or antialigned). When the temperature grows further the magnetic moment in each monolayer (and thus in the whole system) drops to zero at the Curie temperature as determined above. Thus, on the simulated magnetization vs temperature curves one could expect two cusps, one at a lower temperature that corresponds to the breakdown of the antiferromagnetic alignment of the two monolayers, and another one at higher Curie temperature. However, in the simulation data on the magnetization vs temperature, there is no signature of the breakdown of antiferromagnetic configuration. Apparently, it would be resolved in a multilayer system^47^.

**Figure 3:**
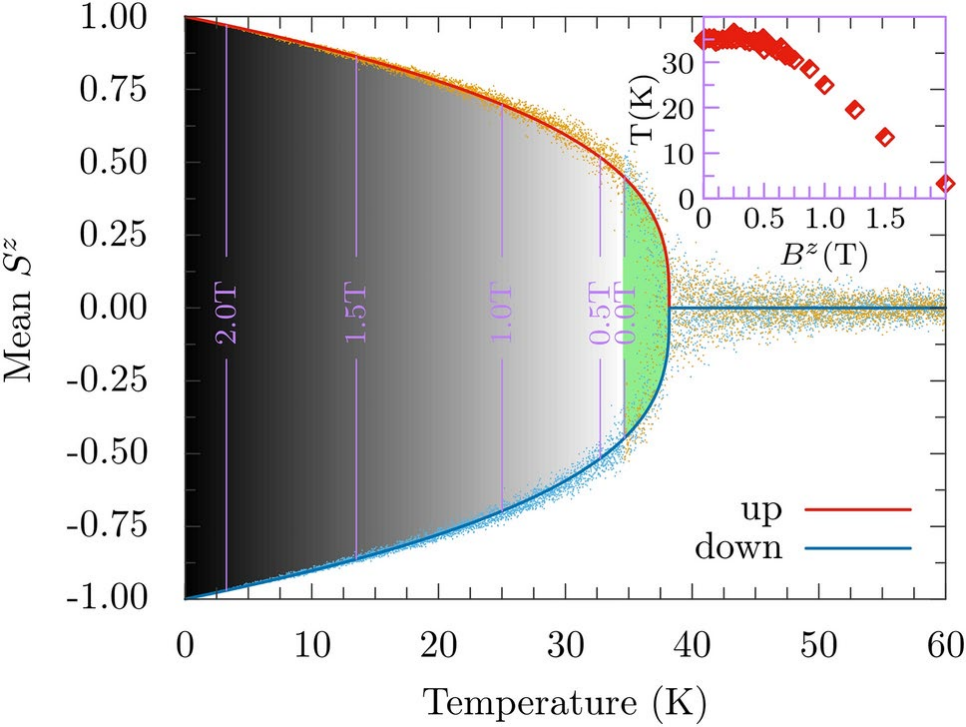
Simulated normalized values of $S^z$ for both monolayers in the antiferromagnetically coupled Rh-stacked bilayer, presented as a function of temperature. These normalized values of $S^z$ for the top and bottom layers are shown as the yellow and blue dots, respectively. The solid red and blue lines indicate the average of $S^z$ for both layers. The grey area between these lines indicates the temperature range where reorientation of the magnetic ordering between the layers does not happen in the absence of external magnetic field. Reorientation begins at the border between the grey and green areas, and the temperature at which this takes place (around 34 K) we assign as the Néel temperature $T_N$. When an external magnetic field normal to the bilayer is applied, the reorientations start at lower temperature, as shown in the inset, and also marked schematically on the grey area. The parameters as in Fig. 2.

In order to get some information on the temperature at which the antiferromagnetic alignment of the two ferromagnetic monolayers is destabilized by thermal fluctuations, we analyzed average values of the total spin *z*-component of each monolayer separately as a function of temperature. Results of the simulations are shown in Fig. 3, where the simulated normalised values of $S^z$ for the two monolayers are presented with blue and yellow dots, while the average values are shown by the solid blue and red lines. With increasing temperature, fluctuations around the average values become larger, and at a certain temperature (recognized as the relevant Néel temperature) the average spin moments of the two monolayers start to change sign. The Néel temperature was identified as a temperature at which spin moments of the monolayers begin to fluctuate between the up and down orientations, see Fig. 3. The corresponding Néel temperature is lower than the Curie temperature by about 3.5 K, as one might expect.

### Hysteresis loops and spin textures

Hysteresis curves are important characteristics as they include information on magnetization variation with increasing/decreasing external magnetic field and thus also on possible field-induced phase transitions. We have determined the hysteresis loops from the Monte-Carlo (MC) simulations as well as from the atomistic Landau-Lifshitz-Gilbert (LLG) equation. The starting point for each simulation was a random state at temperature $T=80\textrm{K}$ and cooling field $H_{\textrm{fc}}=1\textrm{T}$ applied perpendicularly to the sample plane. The field-cooling procedure was used to cool the system’s temperature down to $T=15\textrm{K}$, and then the simulations of a hysteresis loop was initiated. For simplicity and without loss of clarity, in this subsection we simplify the notation for the NN DMI parameters: $d^{NN}_{xy} \equiv d_{xy}$ and $d^{NN}_z \equiv d_z$.

#### Monolayers

**Figure 4:**
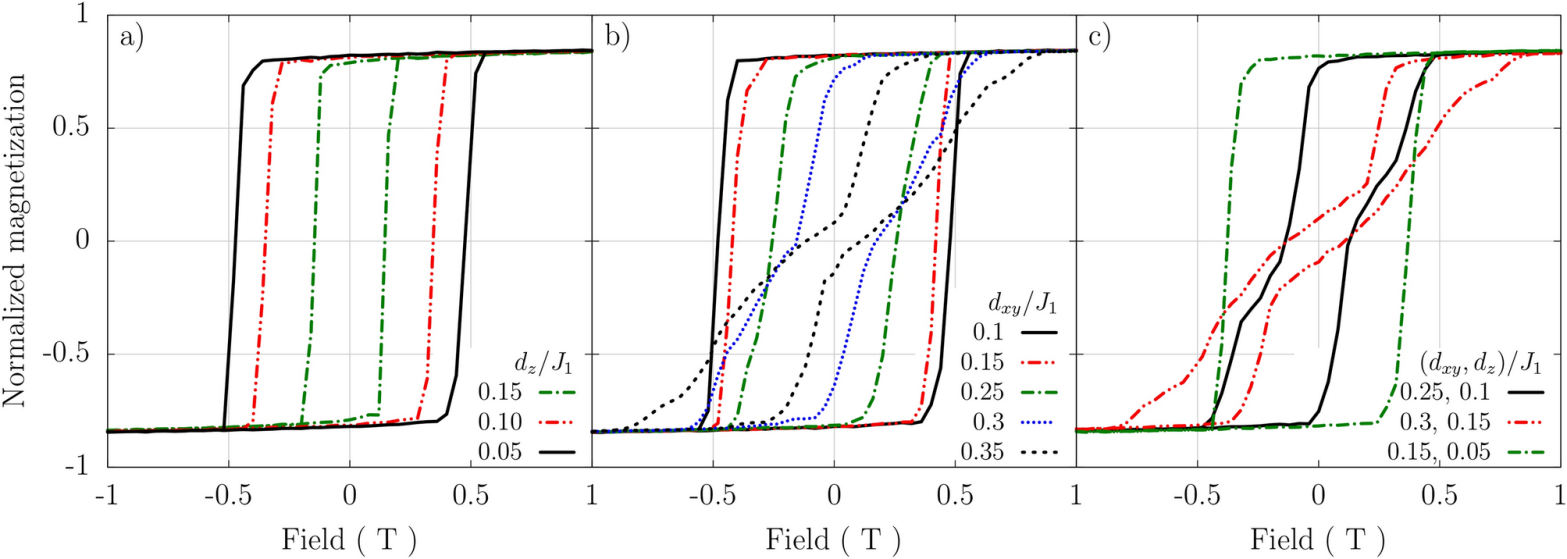
Hysteresis loops for monolayers at $T=15$K, and for the NN DMI parameters: (**a**) $d_{xy}=0$ and $d_z/J_1$ as indicated; (**b**) $d_{z}=0$ and $d_{xy}/J_1$ indicated; and (**c**) $d_{xy}/J_1$ and $d_z/J_1$ as indicated. The other parameters as in the Section “Structure and spin Hamiltonian” and also in the caption to Fig. 2. The external magnetic field is swept along the normal to the layer. The presented results are the averages over 50 realizations.

Figure 4 shows the hysteresis curves for indicated values of the components $d_z$ and $d_{xy}$ of the DMI between NNs. Other parameters, including DMI between NNNs, are given in the caption to Fig. 2. When $d_{xy}=0$, then increasing the component $d_z$ leads mainly to reduced coercivity of the hysteresis curves, while their shape remains typical for ferromagnetic layers, see Fig. 4a. A more interesting impact of DMI on the hysteresis curves appears for the $d_{xy}$ component. The corresponding hysteresis curves for monolayers are shown in Fig. 4b for indicated values of $d_{xy}$ and $d_z=0$. For small values of $d_{xy}/J_1$, the hysteresis curves are qualitatively similar to those for zero $d_{xy}$, but the corresponding coercivity becomes reduced with increasing $d_{xy}$. When $d_{xy}$ grows further, some new features appear in the curves, see e.g. the curves for $d_{xy}/J_1=0.3$ and $d_{xy}/J_1=0.35$, which clearly distinguish these loops from those typical for ferromagnetic layers. The interplay of both components of NN DMI components is presented in Fig. 4c, where both components are nonzero and comparable. For the assumed parameters the corresponding hysteresis loops contain clear features due to both $d_z$ and $d_{xy}$ components of the DMI vector. From the hysteresis curves shown above one may conclude that DMI modifies magnetic texture of the system and this modification leads to the observed features in the hysteresis loops. In the curves shown here, the most pronounced features are for $d_{xy} =0.35J_1$. Therefore we analyse now in more details this specific situation ($d_z=0$ and $d_{xy} =0.35J_1$), and for clarity, we also omit here the intrinsic NNN DMI.

**Figure 5:**
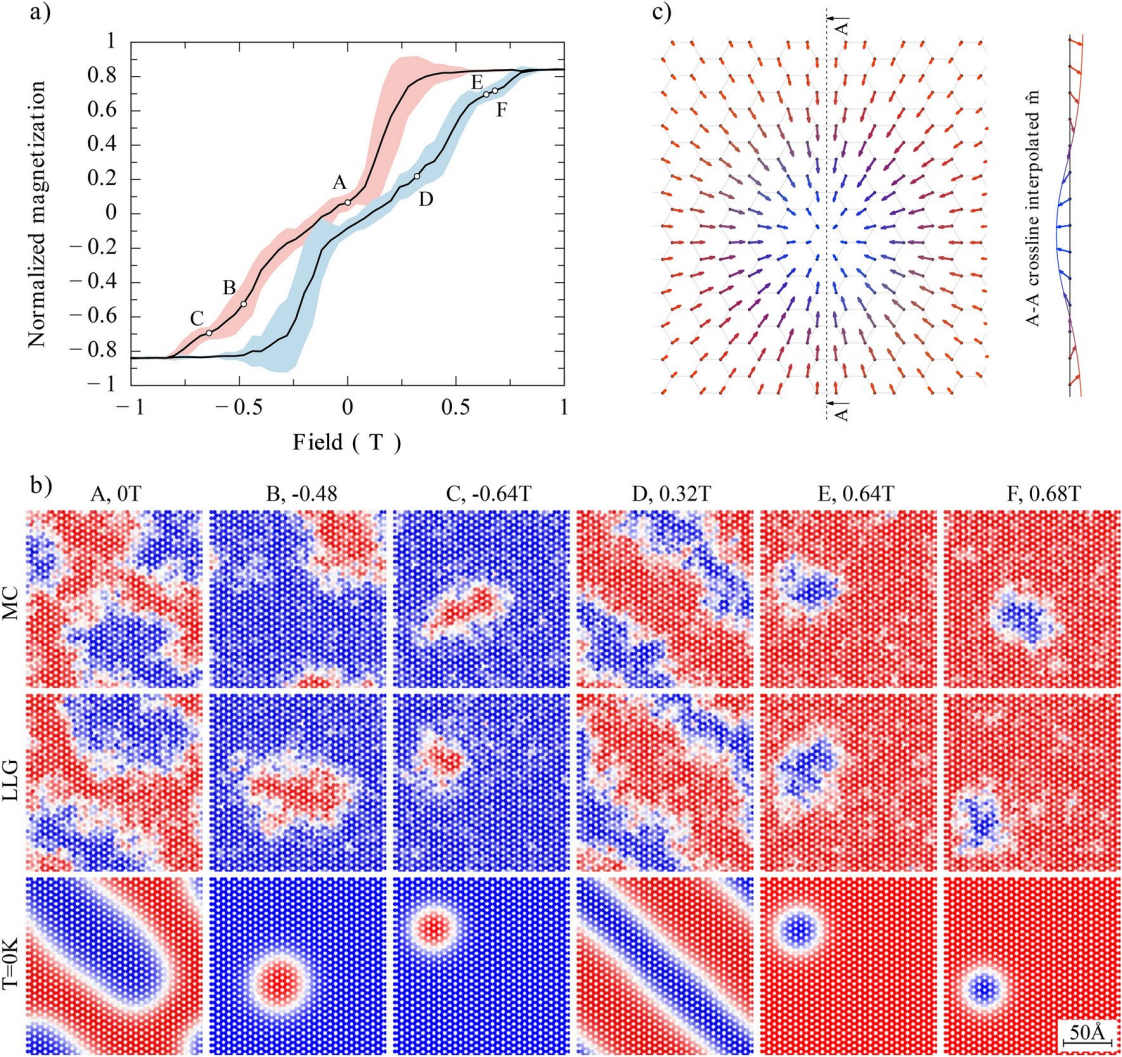
(**a**) Hysteresis loop for the monolayer with $d_{xy} = 0.35 J_1$, $d_z =0$, $T=15$K, and vanishing NNN DMI. The solid black line is the average over 50 realizations, while the shaded area represents the standard deviation. The circle points represent realizations that are closest to the average (solid line), for which the corresponding magnetic textures are shown in part (**b**) of this figure. (**b**) The magnetic textures corresponding to the points A, B, C, D, E and F, marked in the hysteresis loop (**a**). The results of MC simulations are shown in the top row. Then, the final set of MC simulations was used as initial step for simulations based on the atomistic LLG equation, and the middle row shows the magnetic textures after 1ns of spin-dynamics using the LLG Heun method with damping factor $\lambda =0.1$. Then the results from the middle row were used in the next simulation for cooling the temperature from 15K down to 0K. The spin textures upon cooling down to 0K are shown in the bottom row. Upon cooling, the magnetic patterns (stripes, skyrmions) have regular edges. (**c**) Magnified single skyrmion obtained upon cooling the magnetic pattern corresponding to the point F ($B=0.68\textrm{T}$) in (**b**). Here, the spin structure of the skyrmion is clearly resolved. This spin structure also confirms that the created skyrmions are of Néel-type, as presented by the vertical crossection through the skyrmion center, shown on the right side.

In Fig. 5a we show the average hysteresis curve (solid line) and the standard deviation from the average (shaded areas) for $d_{xy} =0.35J_1$, $d_{z} =0$ and $T=15$ K. The corresponding spin patterns for the points A, B, C, D, E and F, indicated on the hysteresis curve, are shown in Fig. 5b.

Here, the results of MC simulations are shown in the top row. Then, the final set of MC simulations was used as initial step for simulations based on the atomistic LLG equation. The middle row shows the magnetic textures after 1ns of spin-dynamics using the LLG Heun method with damping factor $\lambda =0.1$. We have changed the numerical integrator from Monte Carlo to LLG in order to check if the spin texture was stable under both integrators. The results from the middle row were subsequently used in the next simulation for the cooling temperature from 15K down to 0K. As the patterns for $T=15$K have spin textures irregular at the boundaries due to thermal fluctuations, the edges become regular when cooling the system temperature down to 0 K, see bottom row in Fig. 5b. As one can see, upon cooling the edges of various objects become smooth and one can clearly distinguish there regular circular objects and stripe structures. The circular objects are Néel-type skyrmions. To see this in more details, in Fig. 5c we zoomed in an individual circular object, so now the spin structure associated with the skyrmion is clearly visible. To show further details, on the right side of this figure we present a crossection through the center of the skyrmion. The difference in size of skyrmions in the points B and C of Fig. 5b follows from the difference in the corresponding magnetic fields. Similarly, the difference in skyrmion size in the points E and F also follows from the difference in the corresponding magnetic fields, which now is smaller so the difference in the skyrmion size is also smaller. This is in agreement with Ref. ^48^, where the magnetic field dependence of the skyrmion size was investigated.

#### Bilayers

**Figure 6:**
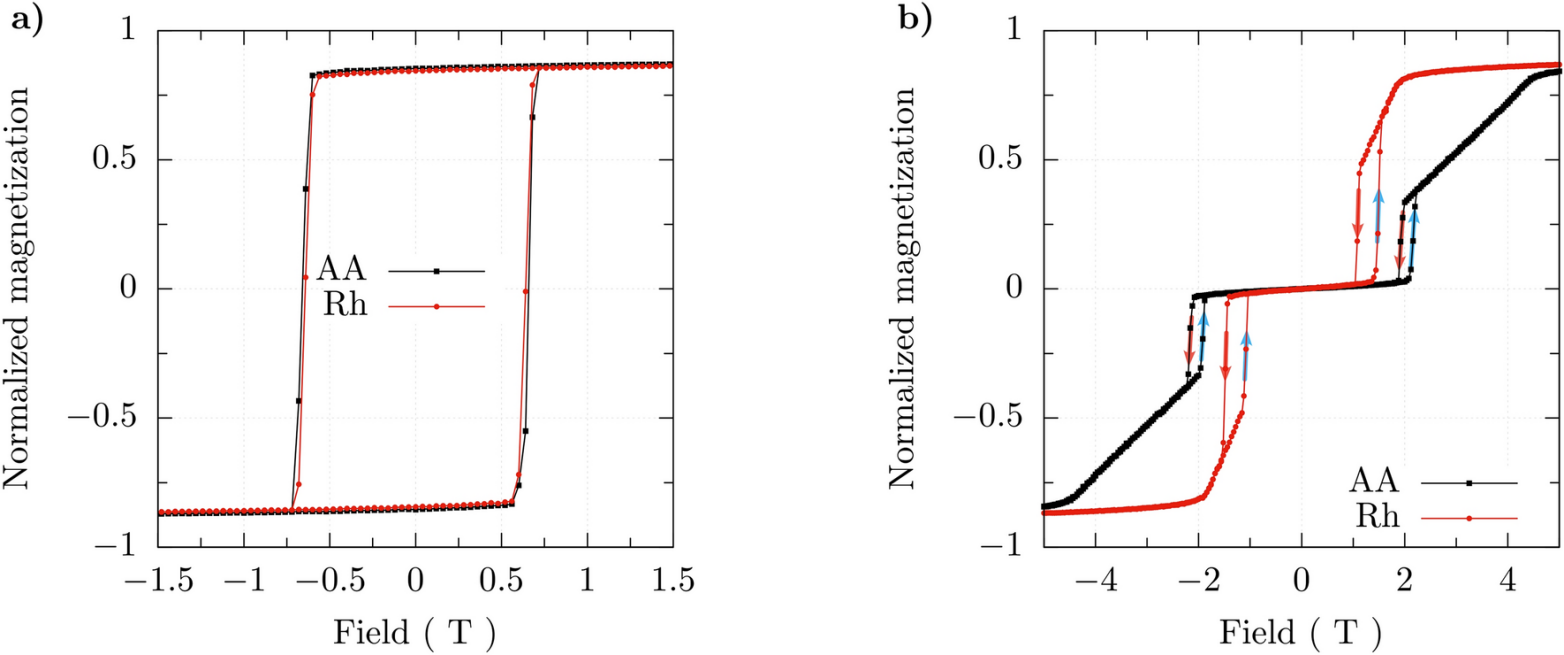
Hysteresis loops for bilayers in the AA and Rh stacking with (**a**) ferromagnetic interlayer coupling, and (**b**) antiferromagnetic interlayer coupling. The hysteresis loops have been calculated for the temperature $T=15$ K and for vanishing all DMI terms, while the other parameters are as in Section “Structure and spin Hamiltonian” and caption to Fig. 2.

Hysteresis loops in bilayers depend of the nature of interlayer coupling, and for ferromagnetic and antiferomagnetic couplings can be remarkably different. In addition, they also depend on the stacking geometry. The hysteresis curves for the AA and Rh stackings in the absence of DM interactions are shown in Fig. 6a for the systems with ferromagnetic interlayer coupling and in Fig. 6b for the systems with antiferromagnetic interlayer coupling.

**Figure 7:**
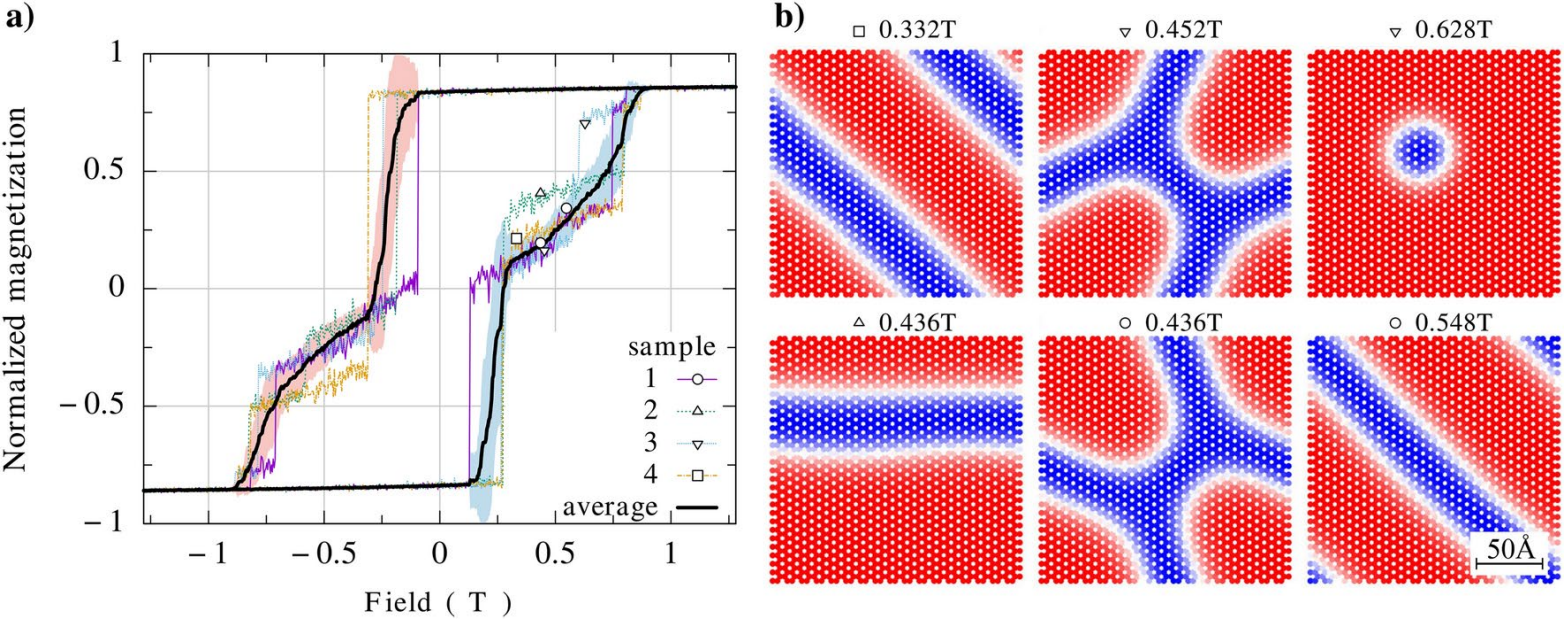
(**a**) Hysteresis loop of a ferromagnetically coupled bilayer in the Rh-stacking, calculated for $d_{xy}/J_1 = 0.35$, $d_z=0$, $T=15$K, and for other parameters as described in the caption to Fig. 2. The solid black line is the averages over 50 realizations, while the shaded area represents the standard deviation. The numbers 1 to 4 indicate four exemplary hysteresis loops obtained in the corresponding numerical simulations. (**b**) Spin patterns in ferromagnetically coupled Rh-stacked bilayer upon cooling, obtained for the points indicated on the hysteresis loop in (**a**). Only spin pattern of the top layer is shown here, as that of the bottom layer is approximately the same. One can distinguish here stripes, skyrmions, and other patterns with regular edges.

**Figure 8:**
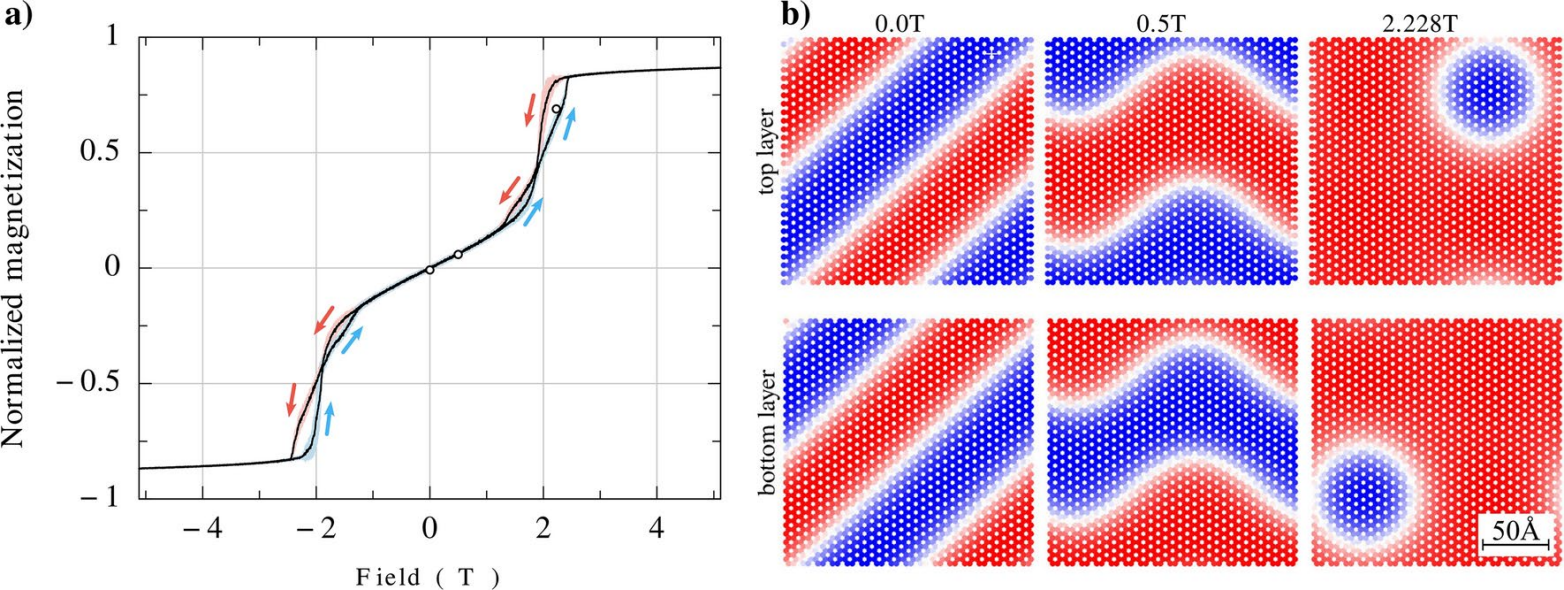
(**a**) Hysteresis loop for antiferromagnetically coupled Rh-stacked bilayer, simulated for $d_{xy} = 0.35 J_1$, $d_z =0$, $T=15$K, antiferromagnetic interlayer coupling $J_z = - J_2$, and NNN DMI as described in the text. The solid black line presents the averages over 50 realizations, while the shadow represents the standard deviation. (**b**) The spin patterns for three values of the magnetic field, including the case with zero magnetic field. These magnetic fields correspond to the three points indicated in the hysteresis loop (**a**).

From this figure follows that the hysteresis curves for ferromagnetically coupled bilayers are similar to typical hysteresis loops of ferromagnetic layers, and are roughly independent of the stacking geometry. On the other hand, the hysteresis curves for bilayers with antiferromagnetic interlayer coupling are significantly different from those for ferromagnetically coupled bilayers, and also different for different stacking geometries. Important feature of the hysteresis loops for antiferromagnetic interlayer coupling is a wide plateau at zero magnetization, which corresponds to antialigned magnetizations of the two monolayers. At a certain value of magnetic field there is a jump in magnetization associated with transition to the spin-flop phase (note, the magnetic field is normal to the layers). Then the system goes smoothly to the saturated state. The magnetic field, at which the transition to the spin-flop phase occurs with increasing magnetic field is different from the field at which the system goes back to the antialigned configuration when the magnetic field decreases. This leads to minor loops, that are clearly visible in Fig. 6b on both sides (positive and negative magnetic fields). These loops follow from the magnetic anisotropy in the system, and also depend on the stacking geometry. No such features appear in ferromagnetically coupled bilayers. The saturation field also depends on the stacking configuration. This is because the saturation field depends on the effective coupling between the two monolayers, and this coupling depends, in turn, on the strengths of interlayer exchange parameter, and on the number of interlayer nearest neighbours. Note, the hysteresis curves for the bilayers in the AA and Rh stacking have been calculated for the same interlayer exchange parameters. However, the numbers of nearest neighbors in the Rh stacking is larger. Thus, for the assumed parameters the saturation fields are different for both stacking geometries, and this field is larger for the Rh stacking.

Let us consider now the impact of DMI coupling on the hysteresis curves of bilayers. Such hyteresis curves in the Rh stacking geometry and for ferromagnetic and antiferromagnetic interlayer coupling are shown in Figs. 7a and 8a, respectively, while the corresponding spin textures are shown in Figs. 7b and 8b. Both NN and intrinsic NNN DMI terms are included in the calculations. As already discussed above, the intrinsic NNNs DMI is rather small in $\hbox {CrI}_3$. In turn, the DMI between NN’s can be easily tuned by external gate and therefore can be dominant (even if it vanishes in pristine $\hbox {CrI}_3$). Therefore, in Fig. 7 and Fig. 8 we assumed intrinsic values of NNN DMI and a quite large value of DMI between NNs, $d_{xy}/J_1 = 0.35$, while $d_z=0$, and focused there on the impact of the $d_{xy}$ component of DMI, as this component is crucial in the skyrmion texture formation.

In the case of bilayers with ferromagnetic interlayer coupling, the spin patterns are shown in Fig. 7b for a few points indicated on the corresponding hysteresis curve, see Fig. 7a. In general, we find there stripes, skyrmions, and also other structures. Only spin patterns of the top monolayer are shown there as the spin patterns of the bottom monolayer are practically the same due to ferromagnetic interlayer coupling. Certain, almost not resolved difference may appear due to offset of the monolayers in the Rh stacking. The skyrmions in both layers are coupled ferromagnetically and have the same topological charge. In turn, in bilayers with antiferromagnetic coupling, the corresponding spin patterns are qualitatively similar to those in the ferromagnetically coupled bilayers, and we find the spin stripes, wavy stripes, and skyrmions, as shown in Fig. 8b. Though they look qualitatively similar to those in ferromagnetically coupled bilayers, they are different and this difference originates from different magnetic fields for which the spin patterns have been simulated and also from opposite signs of the interlayer coupling. In the simulation results shown in Fig. 8b, we show the spin patterns of both top and bottom layers. Note, for small magnetic fields (H = 0 and H = 0.5 T), the spin patterns of both layers are practically opposite due to antiferromagnetic interlayer coupling. The situation is different for stronger magnetic field, H = 2.28 T, which is strong enough to overcome the interlayer exchange coupling. In that case the spin patterns of both layer reveal individual skyrmion in the spin pattern of the top layer and also individual skyrmion in the bottom layer. These two skyrmions, however, are not coupled and appear in different positions.

Finally, we also note here, that the hysteresis loops and the corresponding spin patterns of the monolayers and bilayers, as shown above, are for isotropic exchange interactions. These loops and spin patterns may become remarkably modified when including anisotropic exchange interactions, or more complex anisotropy terms (like a Kitaev term).

### Artificially created Skyrmion lattices

**Figure 9:**
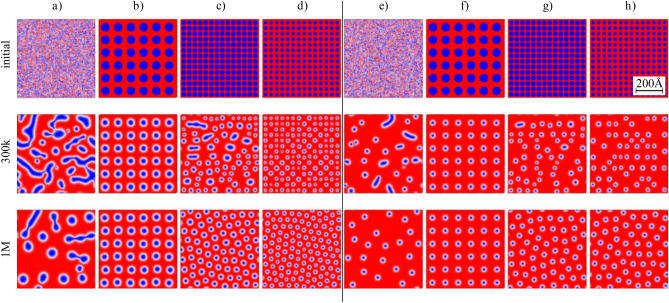
Spin patterns in ferromagnetically coupled Rh-stacked bilayers upon cooling for high density of skyrmions. The left part (the columns (**a**–**d**)) is for *B*=0.3T, while the right part (columns (**e**–**h**) ) is for *B*=0.748T. The top row shows the initial spin configurations: random in (**a**) and (**e**) and arrays of spots with manually reversed magnetizations. The other two rows correspond to the spin structure of the bottom layer after certain number of Monte Carlo steps, as indicated. Parameters: $d^{NN}_{xy}=0.35J_1$, $d^{NN}_z=0$, $J_z = J_2$, while other parameter (including NNN DMI) as in the text and caption to Fig. 2.

In the spin patterns shown above for monolayers, Fig. 5, and for bilayers, Figs.7b and 8b, only single skyrmion states have been found in the corresponding simulation processes for the assumed parameters. Of course, for other parameters, especially for stronger DMI, one can achieve stable multi-skyrmion textures or skyrmion lattices^33,34^. However, we show now that one can create arrays of (quasi)stable skyrmions by appropriate artificial arrangement of initial spin states, obtained by reversing manually the local magnetization at the nodes of a hypothetical lattice, see the top panel in Fig. 9. The parts (a) and (e) of this panel present random initial states, while the other parts show the initial states arranged manually in the magnetic field 0.3T (b–d) and 0.748T (f–h). The evolution of the initial states is analyzed by Monte Carlo simulations, and the second and third rows of these columns show the spin structure after 300,000 and 1000,000 of Monte Carlo steps.

In case (a) we get at the and (after 1M steps) individual as well as coupled skyrmions. In the case (b) the initially created square lattice evolves into a square lattice of skyrmions, with skyrmion size smaller than the size of initially created spots of reversed magnetization. In turn, in case (c) and (d) the system after 1M of simulation steps evolves to smaller skyrmions arranged in a defected hexagonal lattice. The origin of the defects might be twofold. First, they appear naturally, because of the square shape of the simulation cell, which is incompatible with a hexagonal lattice. Second, defects might be excited at increased temperature. Indeed, such disordered states appear in the melting process of a hexagonal skyrmion lattice (skyrmion crystals) with increasing temperature, and the corresponding phase is usually referred to as the hexatic phase^49^. The columns (e) to (h) in Fig. 9 correspond to the same initial states as in the columns (a) to (d), but the simulations are in the magnetic field of 0.748T. In all the four cases (e) to (h), after 1M of Monte Carlo steps we get the arrays of skyrmions (of approximately equal size). Interestingly, the square lattice in column (f) survives after 1M of simulation steps. From the above results one may conclude that the simulated skyrmion lattices may be stable or quasi-stable in time, or can evolve into a disordered hexagonal lattice (hexatic phase). This also shows that one can intentionally create skyrmion lattices, which may be of some interest in skyrmionics. However, their stabilization requires further investigations^50^

### Dynamical properties

Spin waves (magnons) in van der Waals materials are of current interest from both fundamental and practical application reasons^7,37–40^. On one side, this is related to their topological properties, and on the other side, to their possible applications in 2D magnonics. Below we analyze magnon spectra in both monolayers and bilayers of $\hbox {CrI}_3$, and focus on their topological properties induced by the Dzyaloshinskii-Moriya interaction. We note here, that in recent papers^51,52^ we have analysed spinwave modes in monolayers and bilayers of another class of vdW materials, i.e. of Vanadium-based dichalcogenides. These materials have atomic structure and also spin wave spectra that are distinctly different from those of $\hbox {CrCl}_3$. Moreover, properties of spin waves in these two classes of vdW materials are also very different. We also mention, that the method used there was based on quantum-mechanical approach, including Holstein-Primakoff and Bogolubov transformations, while here we use the atomistic simulation technique based on classical equations.

Let us now briefly analyse some features of the spinwave spectra of monolayers and bilayers of $\hbox {CrI}_3$. To obtain the spectra we use the atomistic simulation method implemented in the ASD Uppsala code^46^. Within this approach, dynamics of individual spins is described in terms of the classical Landau-Lifshitz-Gilbert equation. To avoid skyrmion textures, which may occur when DMI is relatively large, we assume zero external magnetic field. Basic features of spin waves in monolayers of $\hbox {CrI}_3$ were already studied in recent years, both experimentally and theoretically^15,53–61^. However, much less attention was paid to spin waves in bilayers^39,58^. As concerns theoretical side, both analytical methods as well as numerical simulation techniques were used to determine the spinwave spectra in $\hbox {CrI}_3$. Generally, the magnon spectra in centro-symmetric systems depend mainly on the exchange integrals and magnetic anisotropy constants. In non-centro-symmetric systems, however, interactions originating from spin-orbit coupling, like Dzyaloshinskii-Moriya coupling and two-ion anisotropy (Kitaev term) play an important role, and are responsible for topological properties of magnons.

**Figure 10:**
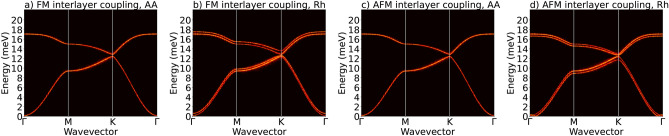
Dispersion curves of spin waves in bilayers with DMI included. The spin wave spectrum for the bilayer in the (**a**) AA stacking with FM interlayer coupling; (**b**) Rh stacking with FM interlayer coupling; (**c**) AA stacking with AFM interlayer coupling; (**d**) Rh stacking with AFM interlayer coupling. These spectra have been calculated for the following NN DMI parameters: $d_{xy}^{NN} = 0.05\, J_1$ and $d_z^{NN}=0$; and for the NNN DMI parameters: $d_{xy}^\textrm{NNN} = -0.478\, \textrm{meV}$, $d_z^\textrm{NNN} = - 0.0576\, \textrm{meV}$. Other parameters are given in Section “Structure and spin Hamiltonian” and also in caption to Fig. 2.

Let us consider first the spinwave modes in bilayers, assuming intrinsic exchange and NNN DMI parameters, as described above in Section “Structure and spin Hamiltonian” and also given in the caption to Fig.  2. For the NN DMI parameters, we assume $d_{xy}/J_1 = 0.05$, which is rather small. Additionally, we also assume $d_z=0$ and neglect Kitaev term. Here, we focus on spin waves in bilayers in the AA and Rh stackings, and with FM and AFM interlayer exchange coupling. The corresponding dispersion curves are shown in Fig. 10 along the $\Gamma - M - K - \Gamma$ path in the Brillouin zone. From earlier works it is known, that there are two modes in a monolayer^7,37–40^. Accordingly, one can expect four modes in bilayers due to splitting induced by the exchange coupling between the two individual monolayers. However, the interlayer coupling is rather small, so the splitting is not well resolved. Indeed, in the case of AA stackings, the splitting of the modes is not resolved for both FM and AFM couplings, Figs. 10a,c. However, the splitting is clearly resolved in the case of Rh stacking, see Fig. 10b,d. This difference follows form stronger effective coupling in the Rh stacking due to larger number of interlayer NNs in the Rh stacking (while the interlayer exchange parameter has been assumed the same for both AA and Rh stackings). However, in all the spectra one can note a small gap in the point K. This gap is induced by the NNN DMI and the spectrum around this point reveals topological properties.

To verify origin of the gap in the spin wave spectrum at the Dirac K point within the assumed model Hamiltonian, we will analyze now the spinwave spectrum in monolayers of $\hbox {CrI}_3$, assuming enhanced values of the NNN DMI parameters. As we have already mentioned in Section “Model and methods”, the intrinsic NNN DMI is rather weak in the pristine $\hbox {CrI}_3$, but it can be remarkably enhanced by proximity effect to a substrate or due to gating. For example, a strong enhancement of DMI can exist, e.g., in the chromium trihalide Janus structures^34^, or due to proximity to a ferroelectric van der Waals material.

**Figure 11:**
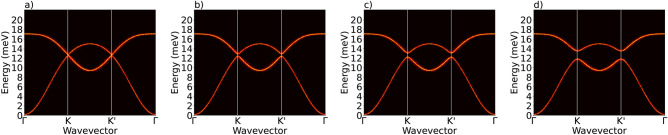
Dispersion curves of spin waves in a monolayer (**a**) without DMI, and (**b**–**d**) with NNN DMI. The DMI parameters used in the calculations: $d_{xy}^\textrm{NNN} = -0.478\times \eta \, \textrm{meV}$, $d_z^\textrm{NNN} = - 0.0576\times \eta \ \textrm{meV}$, and $d_{xy}^{NN} = d_z^{ NN}=0$. The factor $\eta$ is: (**b**) $\eta = 1$, (**c**) $\eta = 2$, and (**d**) $\eta = 4$. Other parameters as given in Section “Structure and spin Hamiltonian” and caption to Fig. 2.

Though the topological features of the spinwave spectrum in monolayers of $\hbox {CrI}_3$ were already studied^15,53–61^, we calculate now this spectrum for four different values of the NNN DMI parameters, focusing on the topological features of the spectrum iduced by the NNN DMI. The results of numerical simulations are shown in Fig. 11 for four different values of the NNN DMI parameters. In the absence of NNN DMI, there is no gap in the spinwave spectrum, Fig. 11a. However, with increasing DMI parameters, the gap opens in the spectrum at the Dirac point K of the Brillouin zone, and width of the gap increases with increasing NNN DMI parameters. Thus, opening of the gap originates here from the Dzyaloshinskii-Moriya coupling. However, it has been shown in several papers on spin waves in $\hbox {CrI}_3$ monolayers, that the Kitaev term has a similar impact on the spin wave spectrum, i.e., it also leads to opening a gap in the Dirac K point. Here we note, that similar atomistic simulation method was also used in our recent paper^62^, were we focused on magnon transport in a different member of the group of chromium trihalides, i.e. in $\hbox {CrCl}_3$. The magnon current was injected there via the spin Seebeck effect and by spin Hall torque, and the main objective was on the dependence of magnon transport on mechanical strain.

## Summary and conclusions

In this paper we have studied magnetic properties of chromium trihalides using numerical simulations within the atomistic spin dynamics and Monte Carlo methods. These simulation techniques have been used to determine critical temperatures, hysteresis curves, spin patterns, and spin wave frequencies. These characteristics have been determined for monolayers as well as for bilayers in two (AA and rhombohedral) stacking configurations, and for ferromagnetic and antiferromagnetic interlayer exchange coupling. The main focus was on the influence of Dzyaloshinskii-Moriya interaction on the basic magnetic properties, especially on the hysteresis curves, spin textures (stripe domains, skyrmion textures, and others), and on the spin wave excitations, where Dzyaloshinskii-Moriya coupling between next-nearest-neighbours leads to opening a gap in the spectrum at the Dirac K point. Both, skyrmion formation and gap opening require relatively strong DMI between nearest-neighbours and next-nearest-neighbours. Accordingly, to observe these features one needs to enhance the DMI either by external electric field (or strain), or due to a proximity effect to a ferroelectric van der Waals material^45^. A strong enhancement of DMI can also appear in chromium trihalide Janus structures^34^.

## Data Availability

The datasets used and/or analysed during the current study are available from the corresponding author on reasonable request.
